# Multiple frequency bioimpedance is an adequate tool to assess total and regional fat mass in HIV-positive patients but not to diagnose HIV-associated lipoatrophy: a pilot study

**DOI:** 10.7448/IAS.16.1.18609

**Published:** 2013-12-27

**Authors:** Patricia Pérez-Matute, Laura Pérez-Martínez, José R Blanco, Valvanera Ibarra, Luis Metola, Mercedes Sanz, Luis Hernando, Sagrario Martínez, Arsenio Ramírez, Enrique Ramalle-Gomara, José A Oteo

**Affiliations:** 1 HIV and Associated Metabolic Alterations Unit, Infectious Diseases Department, Center for Biomedical Research of La Rioja (CIBIR), Logroño, La Rioja, Spain; 2 Infectious Diseases Department, Hospital San Pedro, Logroño, La Rioja, Spain; 3 X-ray Diagnosis Service, Hospital San Pedro, Logroño, La Rioja, Spain; 4 Department of Epidemiology, La Rioja Regional Authority, Logroño, La Rioja, Spain

**Keywords:** HIV-associated lipoatrophy, fat mass, bioimpedance, dual-energy X-ray absorptiometry (DXA), computed tomography (CT scan), diagnostic cut-off values

## Abstract

**Introduction:**

HIV-associated lipodystrophy syndrome causes systemic metabolic alterations and psychological distress that worsen the quality of life of these patients. An early detection should be considered to efficiently treat it. Objective criteria or reference indices are needed for an early diagnosis. Bioelectrical Impedance Analysis (BIA) is an operator-independent, repeatable and non-invasive method of body composition evaluation that is less expensive than dual-energy X-ray absorptiometry (DXA) and/or CT scans. The aims of this pilot study were to validate the data obtained by BIA to measure fat mass in HIV-positive patients with/without lipoatrophy and to determine if BIA correctly diagnoses lipoatrophy in HIV-positive patients.

**Methods:**

Thirty-nine participants were included in this preliminary study. Fourteen were HIV-negative (eight men) whereas 25 were HIV-positive patients (17 men). Eleven of the HIV-positive patients were classified as lipoatrophic according to subjective evaluation by the physicians. Total and regional body composition was measured in basal conditions by DXA and by BIA. To obtain abdominal CT scan fat values, transverse slices with 6-mm thickness were acquired at the L4-L5 intervertebral space.

**Results:**

BIA measurements of total and regional body fat were significantly correlated with those obtained by DXA (*p <* 0.05 to <0.01) in HIV-positive patients. However, agreement between methods was poor as not very high ICC (intraclass correlation coefficient) values were observed. BIA and DXA showed higher ICC values in lipoatrophic patients. The visceral index obtained by BIA was correlated with total and visceral fat in L4 measured by CT scan (*r =* 0.607 and *r =* 0.617, respectively, *p <* 0.01) in HIV-positive patients. The Fat Mass Ratio (FMR) calculated by BIA did not correlate or agree with DXA values.

**Conclusions:**

Multi-frequency BIA could be an effective method to evaluate the evolution of total and regional fat composition in HIV-positive patients with/without lipoatrophy. The correlations between BIA and DXA improved in lipoatrophic patients and in men, suggesting that its efficacy depends on fat mass, gender and probably other factors. The visceral index obtained by BIA seems to be a reliable indicator of abdominal obesity. However, BIA did not fulfil the need for easy quantitative diagnostic tools for lipoatrophy, and it did not provide sufficient diagnostic cut-off values for this syndrome.

## Introduction

Highly active antiretrovial therapy (HAART) has improved the prognosis of HIV-1-positive patients [1, 2]. However, the beneficial effects of HAART have been limited by the development of several side effects. One of the most frequent side effects is the HIV-associated lipodystrophy syndrome (HALS), which is characterized by alterations in adipose tissue distribution in association with metabolic complications [3]. The alterations of adipose tissue consist of central adiposity, peripheral lipoatrophy and lipomatosis, especially in the dorsocervical area (“buffalo hump”). Subcutaneous loss of fat occurs frequently, although more than half of the patients presented with a mixed form: loss of subcutaneous fat together with marked increase in visceral fat [4]. Although the probability of developing HALS has decreased in western countries as HAART prescription has significantly changed, HALS is still a problem [5]. Furthermore, lipodystrophy, especially facial lipoatrophy, can erode self-esteem, cause psychological distress, affect quality of life and lead to depression. It also affects adherence to treatments. To date, there is no effective treatment against lipoatrophy (LD) and the only option is cosmetic surgery.

Development of HALS is slow and clinical detection occurs late [6]. Thus, HALS is clinically evident after 40–50% of limb fat loss, which is rather late to be able to prevent or even treat it. In addition, there is no gold standard method for measuring body fat although several techniques have been used for this purpose such as anthropometry, bioelectrical impedance analysis (BIA), dual-energy X-ray absorptiometry (DXA), computed tomography (CT scan), magnetic resonance imaging (MRI) and ultrasonography. Some of them are expensive and imply radiation [7]. To date, DXA is the recommended method and the most extensively used although it requires training and it is unable to identify facial lipoatrophy [8, 9]. BIA measures the opposition of body cells and tissues to the flow of an alternating electric current. New devices based on BIA have appeared in the market with promising utilities due to their portability and low cost, and because they are non-invasive methods of easy use that do not require specialized facilities or expertise. Previous studies have demonstrated that fat mass measured by BIA is strongly correlated with the data obtained with DXA in a general/control population [10]. There are also some studies where the utility of BIA in HALS patients has been assessed, although the results obtained were controversial [11, 12].

The diagnosis of lipodystrophy is also limited by the absence of an agreed definition and a reference for normality. Accurate diagnosis, especially in mild to moderate cases, is difficult, almost always subjective, non-standardized, and there is no established single method for diagnosis [13]. A quick and easy method to diagnose lipodystrophy will help to prevent or diminish further evolution of this disorder and to evaluate accurately any possible intervention. In 2005, Bonnet *et al*. [14] proposed for the first time an objective method for the definition of HALS: the “fat mass ratio” (FMR). In the following years, different cut-off points have been estimated to diagnose HALS in men and women [15], although there is no agreement about this concern.

The aims of this study were: 1) to compare estimates of total body fat and regional fat (included visceral fat) by BIA with values obtained from DXA and CT scan in HIV-positive patients with and without lipoatrophy; 2) to investigate if the FMR calculated by DXA correlates well with the values obtained by BIA; and 3) to establish the potential reference BIA cut-off values that would help to diagnose lipoatrophy in HIV-positive patients.

## Methods

### Study population

Thirty-nine participants were included in this preliminary study (October 2010–February 2011). Fourteen of the volunteers were HIV-negative (eight men) whereas 25 were HIV-positive (17 men). Eleven of the HIV-positive patients were classified as lipoatrophic according to subjective evaluation by the physicians. Clinical lipoatrophy was defined as peripheral lipoatrophy (changes observed in fat volume in the cheeks next to the nose, lateral aspect of the face, legs, arms and buttocks) without central fat accumulation. All of the candidates that took part in this study had a previous “screening” visit with one physician from the Infectious Diseases Department to ensure that they all met the inclusion criteria. The exclusion criteria for all the volunteers were: suffering from any chronic metabolic or obesity-related disease, hepatic or renal systemic disease such as hypertension, dislipemia, type 1 or 2 diabetes, thyroid function disorders, cirrhosis, fatty liver, and so on; the use of prescription medication that could affect body composition (specially fluid retention), pregnant or lactating women, alcohol or drug abuse (>60 g of alcohol per day or a common consumer of cocaine and other illegal drugs) and usage of metal prosthesis. Previous surgery for lipoatrophy was also an exclusion criterion in HALS.

For each patient, the following information was collected: demographic data (age, gender), HIV infection, AIDS criteria, HIV viral load, duration of infection, type of HAART, HAART duration, CD4 cell counts, and co-infection with hepatitis C or hepatitis B virus. This study was performed following the Helsinki Declaration and was approved by the Committee for Ethics in Clinical Research in La Rioja (CEICLAR). Each patient provided informed consent (Supplementary file).

### Body composition measures

Body composition was measured in the same volunteer and on the same day using a next generation bioimpedance device, a whole body composition DXA and CT scan. All measurements were carried out by specialized personnel and they all took 40 minutes to avoid confusing factors between measurements. Volunteers were cited, fasted and with empty bladder and all measurements were taken wearing light clothes without shoes and socks. All metal objects were removed before the measurements. Height was measured by the same technician to the nearest centimetre in the standing position using a wall stadiometer (SECA, Mexico).

#### Bioelectrical Impedance Analysis

BIA was analyzed using MC-180MA multi-frequency body composition analyzer (Tanita). This method enables the measurement of impedance per segment by switching the points at which the current is applied and the voltage is measured.

Each patient was measured in triplicate to ensure better results and to avoid minimal changes. The mean of these measurements was used for the validation analyses. Each measurement took approximately 1 minute.

#### Dual-energy X-ray absorptiometry

Whole body scans were conducted using a DXA device, model Norland XR-46 (Norland Co, USA; Emsor SA, Spain), by an expert technician. Instrument drift was minimized by daily scanning of DNA phantoms for the quality control of soft tissue composition assessment. Subjects were placed in a supine position and correctly centred on the exploration table. To begin the scan, a starting point was placed 1 cm directly above the centre of the patient′s head and another point was placed in the abdomen (between the iliac blade and the last rib). Hands and feet were also included in the scan. The defined exploration was completed in an average time of 5–10 minutes. Whole and regional fat and fat-free mass were derived using the Norland software.

#### Abdominal CT scan (Somaton, Emotion Duo/6/16 Siemens)

Twelve transverse slices with 6-mm thickness were acquired at the L4–L5 intervertebral space. Analysis of the images and measurement of subcutaneous and visceral abdominal adipose tissue were done by an experienced single observer. Adipose tissue was identified as those areas with pixels between −190 and −30 houndsfields. Subcutaneous adipose tissue was defined as the adipose tissue that circulated the circumference, adjacent to the skin whereas visceral fat was calculated as suggested by Al-Attar *et al*. (2006) [16] due to the similarity observed between the images obtained by MRI and those obtained by CT scan. In order to compare these data with those obtained by DXA and BIA, adipose tissue volume was multiplied by 0.9 kg/L to transform the data into kilograms of fat.

### Statistical analysis

Data are expressed as mean with standard deviations (SD). P-values <0.05 were considered statistically significant. Two different methods (Kolmogorov–Smirnov and Shapiro–Wilk tests) were used to check normal distribution. For the validation outcome, relationships between variables were analyzed by calculating Spearman's rank correlation coefficients. The degree of agreement between measurements was evaluated using the *intraclass correlation coefficient* (ICC) [17, 18]. The grade of agreement is established based on ICC values from 0 to 1 (Supplementary file). We have included both single and average ICC values as only one measurement was taken with DXA whereas the average of three measurements has been used in the case of Tanita MC-180MA. The analysis of receiver-operating characteristic (ROC) has been used to assess the ability of FMR calculated by BIA to diagnose lipoatrophy. Lipoatrophy/lipodystrophy status was set as a FMR ≥1.5 according to DXA [19]. For gender-specific analyses, lipoatrophy/lipodystrophy status was defined as a FMR ≥1.9 in men and ≥1.3 for women [15]. A value with optimal sensitivity and with the highest specificity possible was chosen for BIA [20, 21].

SPSS 18.0 version for Windows (SPSS, USA) and GraphPad Prism 5.0 (Graph-Pad Software Inc., USA) were used for the statistical analyses.

## Results

### Clinical and demographic characteristics of the participants

All of the participants were Caucasian (Table 1). The three groups were very similar in almost all parameters presented although HIV-positive patients were significantly older than controls (*p <* 0.01). HIV groups were also comparable although the number of patients on HAART was significantly higher in the lipoatrophic group in comparison with the non-lipoatrophic group (*p <* 0.05). The duration of the antiretroviral treatment was quite similar between groups.

**Table 1:** Clinical and demographic characteristics of the participants

	Healthy controls *n=*14	HIV-positive patients (no signs of lipoatrophy) *n=*14	HIV-positive patients (with signs of lipoatrophy) *n=*11	ANOVA p (significance)
Men (%)	8 (57.1)	9 (64.3)	7 (63.6)	0.99
Age (years)*	36.2 (7.8)	43.2 (11.6)	49.9 (3.4)	<0.01
Weight (kg)*	70.9 (11.9)	66.2 (12.2)	59.9 (12.3)	0.08
BMI*	24.1 (2.4)	23.8 (3.2)	21.3 (3.5)	0.06
Infection (months)*	–	144.4 (105.1)	214.9 (80.9)	0.07
CD4 count (cells/ul)*	–	783.7 (275.7)	745.4 (403.9)	0.78
HIV RNA load, CV <20 (%)	–	8 (57.1)	8 (72.7)	0.70
Co-infection virus C (%)	–	10 (71.4)	9 (81.8)	0.89
Patients on HAART (%)	–	8 (57.1)	11 (100)	0.04
Accumulative time on HAART* (months)	–	38.7 (34.7)	44.7 (22.2)	0.66
Fat distribution (%) measured by DXA				
Total fat mass*	29 (7.9)	25.9 (8.3)	22.7 (10.3)	0.22
Arm fat*	29.2 (10.6)	25.7 (11.3)	25.8 (12.2)	0.66
Leg fat*	28.1 (8.9)	24.1 (10.6)	19.0 (9.2)	0.07
Trunk fat*	30.4 (8.9)	25.7 (2.7)	23.8 (13.4)	0.29

* (SD).

No differences were observed in total fat mass or fat in arms and trunk between the lipoatrophic and the non-lipoatrophic groups. However, a slight decrease was observed in leg fat in the lipoatrophic group (*p =* 0.07). This difference reached statistical significance when fat was expressed in grams (data not shown).

### Total and regional fat mass measured by BIA and DXA in HIV-positive and -negative men and women

Correlation coefficients obtained when compared to BIA and DXA were higher and more significant in HIV-positive patients than in non-HIV patients. Thus, in HIV-positive volunteers, a very good correlation (*p <* 0.01) between both techniques was found when compared to all fat parameters. A similar tendency was observed in HIV-positive men, whereas only a significant correlation between BIA and DXA was found when measuring total fat mass (%) (*r =* 0.795, *p <* 0.05) and troncular fat (%) (*r =* 0.922, *p <* 0.01) in HIV-positive women. Regarding the agreement between both techniques and that described by the ICC, it is interesting to point out that both BIA and DXA showed higher ICC values when leg fat was measured (moderate concordance for control volunteers: 0.628–0.772 and good concordance for HIV-positive patients: 0.757–0.862) (Table 2).

**Table 2:** Comparison of total and regional fat mass measured by BIA and DXA in HIV-positive and HIV-negative men and women

	Correlation coefficient (Rho)	ICC (individual & average values)
HIV-positive patients (*n=*25)		
Total fat mass (g)	0.739**	0.411–0.583
Total fat mass (%)	0.880**	0.757–0.862
Trunk fat (%)	0.819**	0.593–0.744
Leg fat (%)	0.771**	0.779–0.876
Arm fat (%)	0.779**	0.525–0.688
Fat mass ratio	–	<0.3–<0.3
HIV-positive men (*n*=16)		
Total fat mass (g)	0.806**	0.373–0.543
Total fat mass (%)	0.912**	0.786–0.880
Trunk fat (%)	0.887**	0.681–0.810
Leg fat (%)	0.811**	0.801–0.889
Arm fat (%)	0.796**	0.514–0.679
Fat mass ratio	–	<0.3–<0.3
HIV-positive women (*n*=9)		
Total fat mass (g)	0.657	0.535–0.697
Total fat mass (%)	0.795*	0.665–0.799
Trunk fat (%)	0.922**	0.711–0.831
Leg fat (%)	0.4	0.373–0.543
Arm fat (%)	0.583	0.444–0.615
Fat mass ratio	–	0.249–0.399
Control volunteers (HIV-negative) (*n=*14)		
Total fat mass (g)	0.489 (*p=*0.076)	0.270–0.425
Total fat mass (%)	0.798**	0.523–0.687
Trunk fat (%)	0.059	0.227–0.434
Leg fat (%)	0.686**	0.628–0.772
Arm fat (%)	0.756**	0.440–0.611
Fat mass ratio	–	<0.3–<0.3
Control men (*n*=8)		
Total fat mass (g)	0.762*	0.276–0.433
Total fat mass (%)	0.503	0.611–0.759
Trunk fat (%)	0.749*	0.612–0.759
Leg fat (%)	−0.095	<0.3–<0.3
Arm fat (%)	0.143	0.450–0.621
Fat mass ratio	–	<0.3–<0.3
Control women (*n*=6)		
Total fat mass (g)	0.943**	0.313–0.476
Total fat mass (%)	0.657	0.219–0.360
Trunk fat (%)	0.029	<0.3–<0.3
Leg fat (%)	0.143	<0.3–<0.3
Arm fat (%)	0.371	<0.3–<0.3
Fat mass ratio	–	<0.3–<0.3

* *p<*0.05

** *p<*0.01.

ICC is a descriptive statistic for assessing agreement or consistency between two methods (see ranges in Supplementary file) [22, 23].

Total fat mass (%) (0.786–0.880) and leg fat (0.801–0.889) showed the highest ICC values in HIV-positive men, whereas total fat mass (%) (0.665–0.799) and troncular fat (0.711–0.831) were the highest in HIV-positive women (Table 2). When the control volunteers were classified according to gender, ICC values were very small in almost all measurements, indicating a bad agreement between both techniques. Neither associations nor agreements were found between BIA and DXA when the FMR was calculated.

A significant association between both techniques was observed in all analyzed parameters (*p <* 0.01) in the lipoatrophic group whereas the Rho correlation coefficients were smaller in the non-lipoatrophic group (*p <* 0.05 to <0.01) (Table 3). When the lipoatrophic and the non-lipoatrophic groups were stratified by gender (Supplementary file), the association coefficients were higher and more significant in men compared to women in both groups. In fact, no significant associations were found in non-LD women in any of the parameters analyzed, and only significant associations were observed when measuring total fat mass and leg fat in the LD group (*p <* 0.01). The ICC values were higher in the lipoatrophic group in comparison with the non-lipoatrophic group. A very good agreement was observed when BIA and DXA measured total fat mass (grams and%) (0.906–0.951 and 0.881–0.937, respectively) and good agreements were observed in both troncular and leg fat (0.784–0.879 and 0.722–0.839, respectively) in this lipoatrophic group (Table 3). A similar trend was observed when men and women with signs of lipoatrophy were separately analyzed. Moreover, lower ICC values were observed in leg fat in LD-women in comparison with LD-men (Supplementary file). No associations and no agreements between BIA and DXA were found with the FMR.

**Table 3:** Comparison of total and regional fat mass measured by BIA and DXA in HIV-positive patients with and without signs of lipoatrophy (subjective criteria by physicians)

	Correlation coefficient (Rho)	ICC (individual-average values)
Non-lipoatrophic HIV-positive patients (*n=*14)		
Total fat mass (g)	0.516. *p=*0.059	0.221–0.362
Total fat mass (%)	0.811**	0.603–0.753
Trunk fat (%)	0.732**	0.549–0.709
Leg fat (%)	0.582*	0.835–0.910
Arm fat (%)	0.827**	0.383–0.553
Fat mass ratio	–	<0.3–<0.3
Lipoatrophic HIV-positive patients (*n=*11)		
Total fat mass (g)	0.964**	0.906–0.951
Total fat mass (%)	0.939**	0.881–0.937
Trunk fat (%)	0.945**	0.784–0.879
Leg fat (%)	0.773**	0.722–0.839
Arm fat (%)	0.836**	0.666–0.800
Fat mass ratio	–	<0.3–<0.3

* *p<*0.05

** *p<*0.01.

Similar results were observed when HIV-positive patients were classified into LD and non-LD groups taking into account the 1.5-FMR cut-off [19] (Supplementary file).

### Comparison of troncular fat mass and abdominal fat mass measured by BIA, DXA and CT scan in HIV-positive and -negative men and women

**Figure 1:**
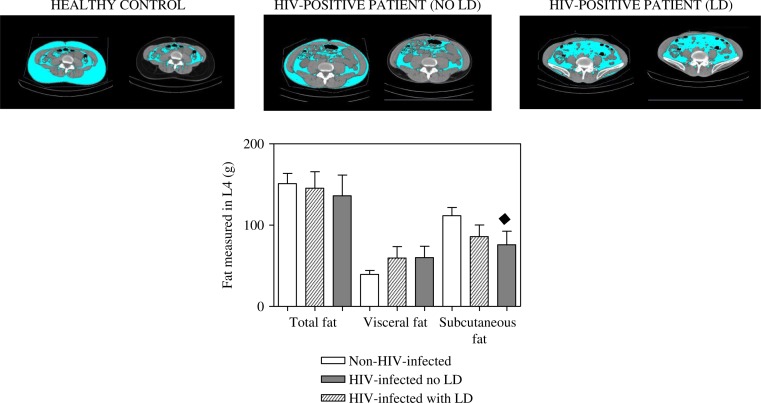
Differences shown by CT scan in total, subcutaneous and visceral fat in L4. Data are expressed as mean±standard error (SE) of at least 11 subjects. ♦ *p =* 0.07 when comparing the values of subcutaneous fat in non-HIV-positive patients (controls) and HIV-positive patients with signs of lipoatrophy (LD).

Visceral fat in L4 was increased in HIV-positive patients in comparison with healthy controls although no statistical differences were observed. A slightly significant decrease in subcutaneous fat in L4 (*p =* 0.07) was observed in HIV-positive patients in comparison with the controls (Figure 1).

Troncular fat measured by BIA (%) was positively correlated with total fat in L4 in both control men (*r =* 0.762, *p <* 0.05) and HIV-positive men and women (*r =* 0.755, *p <* 0.01 for HIV-positive men and *r =* 0.809, *p <* 0.05 for HIV-positive women). Visceral fat index obtained by BIA was positively correlated with trunk fat measured by DXA in HIV-positive men (*r =* 0.77, *p <* 0.01). This visceral fat index was also positively correlated with total and visceral fat (grams) in L4 in controls (*p <* 0.05 to <0.01) and HIV-positive patients (*p <* 0.01) (Table 4). There was no agreement (ICC < 0.3) between BIA and DXA or between BIA and CT scan when the aforementioned parameters were compared (Table 4).

**Table 4:** Comparison of troncular fat mass and abdominal visceral fat measured by BIA, DXA and TC in HIV-positive and HIV-negative men and women

	Correlation coefficient (Rho)	ICC (individual & average values)
HIV-positive patients (*n=*25)		
Trunk fat (%) BIA vs. total fat in L4 (g)^a^	0.730**	<0.3–<0.3
Visceral index vs. trunk fat (%)^b^	0.450*	<0.3–<0.3
Visceral index vs. total fat in L4 (g)	0.607**	<0.3–<0.3
Visceral index vs. visceral fat in L4 (g)	0.617**	<0.3–<0.3
HIV-positive men (*n*=16)		
Trunk fat (%) BIA vs. total fat in L4 (g)^a^	0.755**	<0.3–<0.3
Visceral index vs. trunk fat (%)^b^	0.770**	<0.3–<0.3
Visceral index vs. total fat in L4 (g)	0.716**	<0.3–<0.3
Visceral index vs. visceral fat in L4 (g)	0.560*	<0.3–<0.3
HIV-positive women (*n*=9)		
Trunk fat (%) BIA vs. total fat in L4 (g)^a^	0.809*	<0.3–<0.3
Visceral index vs. trunk fat (%)^b^	0.575	<0.3–<0.3
Visceral index vs. total fat in L4 (g)	0.443	<0.3–<0.3
Visceral index vs. visceral fat in L4 (g)	0.413	<0.3–<0.3
Control volunteers (HIV-negative) (*n=*14)		
Trunk fat (%) BIA vs. total fat in L4 (g)^a^	0.625*	<0.3–<0.3
Visceral index vs. trunk fat (%)^b^	−0.226	<0.3–<0.3
Visceral index vs. total fat in L4 (g)	0.640*	<0.3–<0.3
Visceral index vs. visceral fat in L4 (g)	0.810**	<0.3–<0.3
Control men (*n*=8)		
Trunk fat (%) BIA vs. total fat in L4 (g)^a^	0.762*	<0.3–<0.3
Visceral index vs. trunk fat (%)^b^	0.398	<0.3–<0.3
Visceral index vs. total fat in L4 (g)	0.518	<0.3–<0.3
Visceral index vs. visceral fat in L4 (g)	0.735*	<0.3–<0.3
Control women (*n*=6)		
Trunk fat (%) BIA vs. total fat in L4 (g)^a^	−0.314	<0.3–<0.3
Visceral index vs. trunk fat (%)^b^	−0.213	<0.3–<0.3
Visceral index vs. total fat in L4 (g)	−0.213	<0.3–<0.3
Visceral index vs. visceral fat in L4 (g)	0.334	<0.3–<0.3

* *p<*0.05

** *p<*0.01.

a total and visceral fat in L4 were measured by CT scan

b troncular fat (%) was measured by DXA.

When HIV-positive patients were divided according to gender, correlations observed in total HIV-positive participants were still significant in men, whereas only a significant correlation was found in HIV-positive women (*r =* 0.809, *p <* 0.05) when troncular fat (calculated by BIA) and total fat (estimated in L4 by CT scan) were compared. Troncular fat, visceral index and total and/or visceral fat in L4 measured by BIA, DXA or CT scan correlated better in the lipoatrophic group than in the non-lipoatrophic group (subjective definition or based on FMR cut-offs) (Table 5 and Supplementary file). When non-LD and LD groups were stratified by gender, data obtained in men were comparable than those obtained when men and women were analyzed together. However, no significant associations between techniques were observed in LD-women and only a significant correlation was observed in non-LD women when measuring troncular fat (*p <* 0.01) (Supplementary file). Also, no agreement (ICC < 0.3) was found between all of these values.

**Table 5:** Comparison of troncular fat mass and abdominal visceral fat measured by BIA, DXA and TC in HIV-positive patients with and without signs of lipoatrophy (subjective criteria by physicians)

	Correlation coefficient (Rho)	ICC (individual & average values)
Non-lipoatrophic HIV-positive patients (*n =* 14)		
Trunk fat (%) BIA vs. total fat in L4 (g)^a^	0.618*	<0.3– < 0.3
Visceral index vs. trunk fat (%)^b^	0.371	<0.3– < 0.3
Visceral index vs. total fat in L4 (g)	0.520	<0.3– < 0.3
Visceral index vs. visceral fat in L4 (g)	0.659*	<0.3– < 0.3
Lipoatrophic HIV-positive patients (*n =* 11)		
Trunk fat (%) BIA vs. total fat in L4 (g)^a^	0.821**	<0.3– < 0.3
Visceral index vs. trunk fat (%)^b^	0.720*	<0.3– < 0.3
Visceral index vs. total fat in L4 (g)	0.704*	<0.3– < 0.3
Visceral index vs. visceral fat in L4 (g)	0.566 (*p =* 0.069)	<0.3– < 0.3

* *p <* 0.05

** *p <* 0.01.

a measured by DXA

b troncular fat (%) was measured by DXA.

### Fat mass ratio threshold: ROC curve

**Figure 2:**
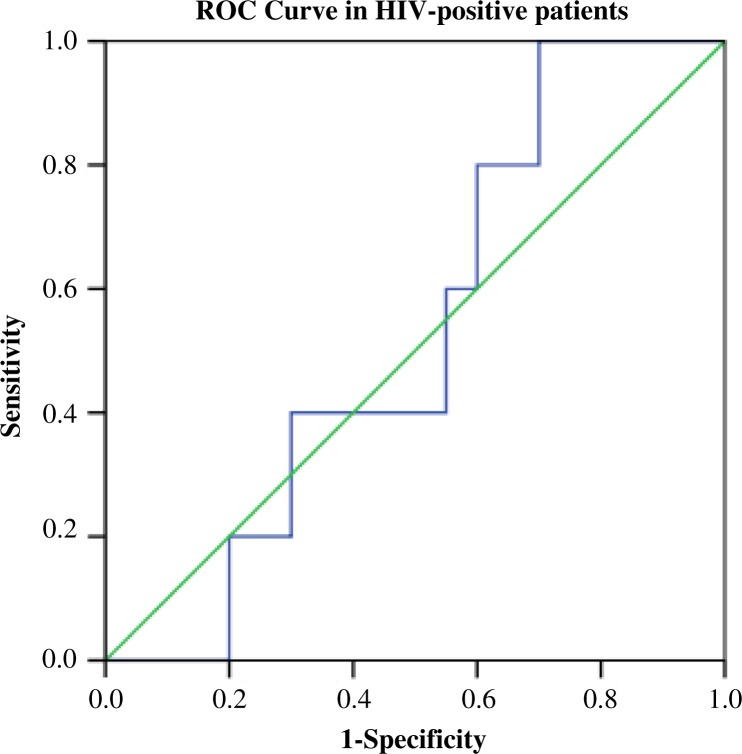
Receiver-operating characteristic curve (ROC) for the comparison between the fat mass ratio (FMR) measured by DXA and the FMR calculated by BIA in HIV-positive patients (both men and women analyzed together). DXA cut-off value (reference value) has been obtained from the study of Degris *et al*. 2010 [19].

FMR calculated by BIA slightly correlated with visceral index calculated by BIA (*r =* 0.320, *p <* 0.05). Considering lipoatrophy/lipodystrophy status as a FMR ≥1.9 in men (*n =* 16), the optimal cut-off values for the FMR calculated by BIA was 1.13 with a sensitivity of 33% and a specificity of 69%. It was not possible to find out cut-off values in women, since only one patient had a FMR >1.3. Thus, we analyzed men and women together in an attempt to increase the sample size. We used an averaged DXA FMR cut-off previously published [19]. Considering lipoatrophy/lipodystrophy status as a FMR *≥*1.5, the optimal cut-off value for the FMR calculated by BIA was 0.70 (AUC: 0.530) with a sensitivity of 80% and a specificity of 40% (Figure 2).

## Discussion

In this study, HIV-positive lipoatrophic patients were defined based on subjective perception by the physicians. All of the lipoatrophic participants presented loss of fat in limbs, whereas no visual accumulation of fat in the abdomen was observed. The L-4 scans demonstrated that total fat mass did not vary between the non-lipoatrophic and the lipoatrophic patients and only a slightly significant decrease of subcutaneous fat was observed in lipoatrophic patients and only when compared with controls. Leg fat depot has been described as the depot most affected in HIV-infection, more specifically in LD [12, 24–27]. The subjective perception of fat loss in limbs was corroborated by DXA measurements, as a decrease in leg fat was observed in the LD group in comparison with the non-lipoatrophic patients. However, this decrease measured by DXA did not reach statistical significance, which suggests that the physician's perception of fat loss should be considered as a warning to control the evolution of that patient in the future.

Our results showed significant correlations (measured by Spearman Rho coefficient) between BIA and DXA when measuring total and regional fat mass in HIV-positive patients. These data confirmed previous studies that suggested that BIA could be used for routine assessment of total body fat mass in HIV-positive male subjects [12, 28–30]. Previous studies demonstrated that the accuracy of BIA in people with/without HIV depended on gender [31]. These studies support the differences observed between men and women in our study. However, it is also tempting to suggest that the smaller correlation coefficients and agreement between BIA and DXA observed in women in comparison with men could also be explained by the smaller number of female participants, especially evident when LD and non-LD HIV-positive people were stratified by gender (four and five subjects, respectively). In this sense, the Rho and ICC values obtained when women were separately analyzed could make it difficult to provide reliable conclusions, mainly due to the small sample size. Thus, we believe that the results with men and women analyzed together are more reliable and give a general idea of the agreement between the techniques in HIV-positive patients, which is the purpose of this study. It is also worth mentioning that the magnitude of Rho correlation coefficients and ICC values were also higher in the LD than in the non-LD patients despite the fact that the number of LD participants were a bit smaller than the non-LD ones (11 vs. 14), suggesting that the accuracy of BIA not only depends on the number of participants but also on gender and/or fat quantity/distribution, as previously suggested [29]. Thus, our results suggested that less fat positively affects the accuracy of BIA as no differences were found when measuring total and regional fat by BIA and DXA in the LD group (Supplementary file). In this context, the study of Bonevas-Asiova *et al*. (2007) also demonstrated that BIA's usage is of limited value in very obese individuals (large fat depots) and may lead to wide individual errors [10].

The poor ICC values observed suggested that BIA could be an easy and useful tool to give us a bigger picture of body composition in both a general and HIV-positive population with/without lipoatrophy and even to evaluate the evolution of fat mass. However, it seems not to be very useful to properly quantify fat mass, which means that it will not be very useful to provide cut-offs to properly diagnose lipoatrophy or other diseases based on fat mass distribution. It is also possible that a statistical artefact could cause the low ICC values obtained, since the ICC is dependent on the variability of the observed values. Thus, if measurements were very similar and therefore they did not vary a lot, the ICC tends to be low [18].

FMR is proposed as an objective method for the definition of lipodystrophy in HIV-positive patients. Different FMR cut-off values for men and women have been proposed [15]. Thus, we analyzed our data separately in men and women using the cut-offs provided by Freitas *et al*. [15, 30]. It was not possible to find out cut-off values in women, since only one patient had a FMR >1.3. Considering lipoatrophy/lipodystrophy status as a FMR ≥1.9 in men, an unusable value with a very low sensitivity (33%) was obtained, probably due to the small sample size. Thus, we decided to analyze men and women together with an average FMR cut-off that has been accepted by previous studies: 1.5 [19]. A different threshold with a better sensitivity and specificity was obtained. It is obvious that the different cut-off values obtained in men when analyzed together with women reinforce the need for gender-specific cut-off values; however, we were not able to perform gender-specific analyses (ROC curves) using the Tanita device mainly due to the small sample size. Our results also suggest that 1.5 is a good approximation for our pilot study as when we classified HIV-positive patients into LD and non-LD groups based on this FMR value, similar correlation coefficients and ICC values were obtained than those achieved with a subjective classification of LD from the physicians. These results underline that the subjective perception of LD by the physicians is a reliable indicator of fat mass redistribution in HIV-positive patients that could be an alternative to diagnose this syndrome in those countries where DXA images are difficult to obtain.

In addition, our study strongly suggests that FMR is only valid for DXA measurements as neither correlation nor agreement was found when comparing the thresholds obtained with DXA with those calculated by BIA. Furthermore, FMR definition was based on lipodystrophic state, which implies both loss of fat and/or increased visceral fat, and in our study only lipoatrophy has been evaluated.

Central adiposity, particularly visceral adiposity, has been linked with metabolic disturbances such as HALS. Novel DXA software has recently been developed to measure visceral fat from a DXA whole body scan. However, this software is very expensive and a very recent study suggests that DXA measurements of abdominal fat are more useful in obese individuals than in non-obese or even anorexic people [32]. This should be taken into account when measuring abdominal visceral fat in HALS. Thus, CT scan and/or MNR (expensive and invasive) are considered the gold standard techniques to quantify abdominal visceral fat. The model of BIA used in this study was able to provide an indirect value of abdominal visceral fat known as *visceral index*. According to manufacturer instructions (based on data from the University of Columbia and Tanita Institute), values >13 indicate a risk of visceral obesity and related disorders such as cardiovascular events. Thus, we have validated for the first time an indirect index for assessing abdominal visceral fat calculated by BIA using CT scan as reference tool. Our results demonstrated that visceral index correlated very well with total fat and visceral fat in L4 measured by CT scan and also, but with a lower significance, with troncular fat measured by DXA in HIV-positive patients. These results suggest that this index could be an adequate tool to evaluate abdominal obesity and therefore could be very useful to diagnose it in an easy, cost-effective and quick way. Our results also demonstrated that the estimation of visceral fat by BIA was not in agreement with the quantity of fat measured by CT-L4 scan, which makes sense since the visceral index is an indirect parameter and the values obtained are arbitrary units.

There are obviously several limitations in this study. One is the small number of subjects, which could limit the generalization of the results. However, there are other validation studies with a very similar number of participants where similar findings have been observed [12], which reinforces our conclusions. We designed this study as a preliminary project and due to the high correlation coefficients observed when compared BIA with DXA and CTscan, we decided it was not needed to recruit a larger cohort for this purpose and, therefore, it was not needed to expose more volunteers to X-ray radiation. In addition, our results are not comparable to an aged HIV-positive population (suggested to have more accumulation of fat mass), as several studies have demonstrated that the accuracy of BIA is affected by the quantity of fat mass [10, 29]. Thus, other studies will be needed in this regard.

Although a disparity of age was observed between the groups (especially evident when the controls and the HIV-positive groups were compared), this study was not designed with a sufficient sample size for an elaborate multivariate model. The main objective of this study was to validate BIA measures in an HIV-positive population as controversial data were published. In this context, no significant differences on age were observed between the lipoatrophic and non-lipoatrophic patients; thus, the divergences observed between both groups should not be influenced by age. The median age in our HIV-positive patients was around 40–45 years. To avoid the interference that menopause as well as other factors such as medications could induce in body fat composition, multivariate analyses will be needed but due to the small size of the groups, we were not able to perform the same.

Another potential limitation of this project is that there are several sources of variance in BIA measurements (blood viscosity, albumin concentration, etc.) that may affect the whole-body impedance measurements. In order to reduce these confounding factors, measurements were also carried out at 8.15 am with an empty bladder and fasted. This BIA device does not respect Espen's guidelines because electrode placement is not standard. This should lead to conduction errors, particularly with subjects with callosity. However, callosity was not observed, and the electrodes were always placed by the same person in accordance with the manufacturer instructions. Finally, body fat *per se*, because it is not conductive, is not measured by BIA. This is a limitation of all two-compartment models where fat is defined as the difference between body weight and free fat mass [11]. However, DXA has measurement error in itself [33] and despite this fact, it is used as a gold standard technique.

## Conclusions

In summary, this is one of the first validation studies carried out in Caucasian people where the data obtained by a novel multi-frequency bioimpedance device have been compared with two gold-standard techniques usually used for body fat quantification in HIV-positive patients. Our results suggested that multi-frequency BIA is a non-invasive, quick/easy and low-cost method that could be an effective and practical method to assess and evaluate the evolution of total and regional fat composition in HIV-positive patients with/without lipoatrophy, as it performed well compared to DXA. The correlation between both techniques improved in lipoatrophic patients and in men, suggesting that the efficacy of BIA depends on fat mass, gender and probably other factors. The visceral index obtained by this device seems to be a non-invasive and reliable indicator of the quantity of visceral fat in the abdomen and therefore an indicator of abdominal obesity and its metabolic consequences. However, the FMR calculated by BIA and BIA itself does not seem to provide specific cut-offs to objectively define HIV-related lipodystrophy/lipoatrophy. Therefore, the usage of BIA for diagnosing lipodystrophy is limited and should be taken with caution.
